# On the Employment of Apiol in Amenorrhœa and Dysmenorrhœa

**Published:** 1866-05

**Authors:** 


### On the Employment of Apiol in Amenorrhœa and Dysmenorrhœa.

BY DR. CORLIEU.

[Apiol is the active principle of parsley. A work has been lately published by Dr. Marrote of the Hospital La Pitié, on the utility of this principle. Dr. Corlieu has now employed it for eight years, and in this paper points out the cases in which it may be expected to prove useful.]

A. In all cases where the menstrual disorder depends upou the derangement of a vital element, where there is plethora or anæmia, apiol should not be used, for, being a nervous tonic, it will only aggravate the condition of the patient. But if the condition of chlorosis be removed, apiol may be prescribed with a good prospect of success. The following case will illustrate this:—A lady, thirty-eight years of age, of a lymphatic and nervous temperament, had suffered for three months from amenorrhœa, complicated with extreme chlorosis. Dr. Galligo at first ordered apiol, but without success. At a later period he combined it with chalybeates, which had previously done no good. The combined use of iron and apiol effected a cure. Dr. Marrote relates the following case:—Miss C., eighteen years old, was of a lymphatic temperament. In childhood she had had measles, hooping-cough, and modified small pox. Her skin was of a dead white, her face somewhat swelled; the gums were swelled and discolored; she had very little appetite, and often vomited her food. She menstruated first when fourteen years old; for several months the discharge was white, afterwards it became a redish color, but was accompanied by such severe uterine pains, that she was obliged to keep her bed. As she was to menstruate on the 18th of October, I ordered her two capsules of apiol on the 15th, two on the 16th, and two on the 17th. On the 18th, the menses appeared, though still in small quantity, but unaccompanied by colics or uterine pains; they only lasted two days, and the blood was still very pale. On the 21st, I ordered a chalybeate which was continued till the 18th of November, when she resumed the apiol for three days. The menstruation was unaccompanied by pains; it continued three days, and the discharge was more colored and more abundant.

B. When the menstrual disorder depends upon a diathetic condition (dartres, scrofula, etc.,) we must, by means of a specific treatment, such as bitters, cod liver oil, preparations of iodine, sulphur, or arsenic, attack the principal malady. Apiol is of no use at first in these cases; but when the cure of the morbid diathesis has been effected, it may be employed with advantage in stimulating the torpid menstrual function.

C. But it is chiefly in disorders which are under the influence of the nervous system that apiol is a heroic remedy, leaving far behind it all the emmenagogues hitherto employed. As a neurotonic it supplies to the nervous system the energy it has lost. Change of life, of habits, or of climate, often determine amenorrhœa. This is a fact which must not be forgotten, and which is well known to the physicians of boarding-schools and religious houses. This menstrual suppression is transitory; it lasts some months, and sometimes only gives rise to slight nervous disorders, or a slight oddity of character. In these cases, two, four, or at most six capsules of apiol will restore the menstrual flux.

The sudden application of cold during a menstrual period may suppress the discharge abruptly, and give rise to amenorrhæa, which may last for an indefinite time. In the month of January, 1861, I saw a young lady, seventeen years of age, who had menstruated for two years, but in whom, in consequence of a chill during menstruation, the flow was suppressed. The belly became considerably enlarged; there was, in fact, an ascites, which could only be explained by the amenorrhœa. There was no albumen in the urine. I employed, without success, purgatives, sudorifics, chalybeates, and the ordinary emmenagogues. There was considerable pain at what should have been the menstrual periods. This state continued until the end of April. In May, capsules of apiol given night and morning restored the discharge, though at first it was pale and serous. Iron was continued, and from that time the abdomen diminished in size. The cure was complete.

It would be easy to bring forward more cases, but the above may suffice. The point I wish to establish is, that apiol is the best emmenagogue with which we are acquainted in all cases where amenorrhœa or dysmenorrhœa have their origin in a disturbance of the nervous element. The principal condition for success in the use of apiol is in the choice of the proper moment for its administration. In almost all cases of amenorrhœa or dysmenorrhœa which depend upon an organic cause, the use of apiol is contraindicated. This is not the place to lay down the different diagnosis of these conditions. If apiol has succeeded in some cases of plethora, it has been because the plethora was not very considerable. "In order," says Dr. Marrote, "that apiol may succeed, it is an essential condition that the pain which accompanies menstruation depend upon dysmenorrhœa, properly so called, that is, on the vaso-motor innervation of the womb. It has never succeeded in calming nervous pains, dull or acute, which were seated in branches of the lumbo-sacral nerves, and especially in the uterus, pains which appear or become exaggerated at the menstrual period, and may, at first sight, simulate dysmenorrhœa proper." Another condition for success in the use of apiol consists in choosing a time for its administration corresponding to a menstrual period. If the woman has not properly calculated the period, we may be enabled to discover it by noticing the sympathetic derangements which occur under these circumstances.—*Gazette des Hopitaux—Edinburgh Medical Journal, August,* 1864, *p.* 164—*Braithwaite's Retrospect.*

